# Dengue Virus 1 Outbreak in Buenos Aires, Argentina, 2016

**DOI:** 10.3201/eid2310.161718

**Published:** 2017-10

**Authors:** Estefanía Tittarelli, Silvina B. Lusso, Stephanie Goya, Gabriel L. Rojo, Mónica I. Natale, Mariana Viegas, Alicia S. Mistchenko, Laura E. Valinotto

**Affiliations:** Hospital de Niños “Ricardo Gutiérrez” Buenos Aires, Argentina (E. Tittarelli, S.B. Lusso, S. Goya, G.L. Rojo, M.I. Natale, M. Viegas, A.S. Mistchenko, L.E. Valinotto);; CONICET, Buenos Aires (E. Tittarelli, S. Goya, M. Viegas, L.E. Valinotto);; Comisión de Investigaciones Científicas de la Provincia de Buenos Aires, Buenos Aires (A.S. Mistchenko).

**Keywords:** Dengue virus, phylogeny, Argentina, genotype V, 2016 outbreak, Buenos Aires, viruses, vector-borne infections

## Abstract

The largest outbreak of dengue in Buenos Aires, Argentina, occurred during 2016. Phylogenetic, phylodynamic, and phylogeographic analyses of 82 samples from dengue patients revealed co-circulation of 2 genotype V dengue virus lineages, suggesting that this virus has become endemic to the Buenos Aires metropolitan area.

Dengue virus (DENV) is a single-stranded RNA flavivirus primarily transmitted among humans by the *Aedes aegypti* mosquito. A substantial increase in dengue incidence has been observed in the past 2 decades in the Americas, and most of Argentina’s bordering countries have reported co-circulation of >1 dengue serotype (*1*). In Buenos Aires, Argentina, the presence of the *A. aegypti* mosquito vector has been reported since 1995; DENV-1 local transmission was detected for the first time in 2009. No new autochthonous cases were detected again until 2016, when the worst dengue outbreak in decades occurred in the Americas (*2*,*3*).

## The Study

During December 2015–April 2016, we confirmed 2,306 cases of human infection with DENV-1 in the virology laboratory at Hospital de Niños R. Gutiérrez. Most (84.69%) cases occurred during February and March. Patient ages ranged from 0 to 93 years (median 30 years). Of the 2,306 laboratory-confirmed cases, 76.7% of patients reported no recent travel history outside the Buenos Aires metropolitan area within 15 days before the onset of fever (local cases). The remaining cases had recently returned from dengue-affected areas (imported cases).

To characterize the outbreak, we sequenced the DENV-1 envelope glycoprotein (E) gene of 82 positive samples from local and imported cases (Technical Appendix Table). We used 3 phylogenetic inference methods that determined that the 82 sequences belonged to DENV-1 genotype V (Technical Appendix Figure 1). We detected 145 mutations in 143 polymorphic sites, and we found 42 sites that were negatively selected with >2 of the assayed methods. We found no positively selected sites. The selection analysis resulted in an overall dN/dS of 0.05, which is consistent with our previous work on the 2009 outbreak sequences, where the overall dN/dS ratio was also <1 (*4*).

We found 18 amino acid substitutions in the E protein. Using the Meta-CATS (metadata-driven comparative analysis tool for sequences) statistical analysis tool (*5**)*, we found that 4 of these substitutions (S338L, R394K, V428L, and V436I) divided our sequences into 2 groups (p<0.01): 1 was related to the 2009 Buenos Aires outbreak and the other to sequences from Brazil (2010–2013). Additionally, we found within the Brazil group a subgroup of 10 sequences containing unique substitutions D235E, K325R, and K361R (p<0.01). Amino acid substitutions are not located on reported epitope positions. Glycosylation sites Asn-67 and Asn-153 were conserved in all the sequences we analyzed.

We performed phylodynamic and phylogeographic analyses on a total of 198 DENV-1 genotype V E-protein gene sequences from the Americas (82 obtained in this study and the rest retrieved from the NCBI Dengue Virus Resource) to analyze the origin, dynamics, and temporal-spatial diffusion process of the 2016 outbreak. We inferred that the most recent common ancestor was located in the Caribbean, with the highest state probabilities in British Virgin Islands (0.54) and Puerto Rico (0.35), by the end of 1975 (95% HPD 1972–1979). The mean rate of nucleotide substitution was 6.95 x 10^-4^ substitutions/site/year (95% HPD 5.87 x 10^-4^ - 8.11 x 10^-4^), similar to previous reports (*6*–*8*).

A maximum clade credibility tree revealed the co-circulation of 2 lineages in Buenos Aires during 2016, characterized by the 4 amino acid substitutions described (Technical Appendix Figure 2). One of the lineages has an inferred origin in Venezuela around 1999 (95% HPD 1998–2004) and arrived in Argentina around 2007 (95% HPD 2006–2007). We found 43 sequences from the 2016 outbreak, along with sequences from the 2009 outbreak previously described by our laboratory, in this lineage. The inferred origin for the second lineage is the British Virgin Islands around 1984 (95% HPD 1982–1985), later arriving in Brazil around 1999 (95% HPD 1996–2000). This lineage comprises 39 sequences from the 2016 outbreak, arranged in 3 subclades originated during 2012–2015.

## Conclusions

Our continuous work in DENV diagnosis, surveillance, and research enabled us to characterize the serologic status of the population of Buenos Aires in 2009. We found that an unusually high percentage of the population had secondary DENV infections in what was considered at the time a nonendemic area; therefore, we proposed that cryptic DENV circulation causing inapparent infections might be affecting this area (*9*). We also described the phylogenetic and phylogeographic characteristics of the first DENV-1 outbreak in 2009; the circulating virus clustered in a monophyletic group within genotype V, which is the most predominant DENV-1 genotype in the Americas (*10*). In this study, we found that the virus in the 2016 outbreak is also genotype V DENV-1; surprisingly, phylogenetic studies revealed that 2 lineages were circulating concurrently. Both identified lineages are related to sequences from different neighboring countries, and we observed no monophyletic groups local to Buenos Aires or other provinces of Argentina. The co-circulation of 2 DENV lineages was recently reported in Brazil (*11*,*12*).

Our data suggest that DENV-1 is established in Buenos Aires and that this densely populated area is changing from one with sporadic outbreaks to an endemic zone. Of note, other arboviruses transmitted by the same mosquito vector, such as Zika and chikungunya, caused autochthonous cases in northern provinces of Argentina in 2016. We believe that the Buenos Aires metropolitan area is now a susceptible area for the emergence of other DENV serotypes, as well as other viruses transmitted by the same vector. Public health authorities should develop stronger prevention and control strategies to avoid future arbovirus outbreaks.

## Ethics Statement

These results are part of a study approved by the Medical Ethics and Research Committees of “Ricardo Gutiérrez” Children’s Hospital, Buenos Aires, Argentina (IRB No. 10.46). We did not obtain informed consent because patient information was anonymized and deidentified before analysis.

Technical Appendix Additional information about the sequencing and analysis of dengue virus genomes.

## References

[1] Pan-American Health Organization/World Health Organization. Five-fold increase in dengue cases in the Americas over the past decade. 2014 [cited 2015 Jul 29]. http://www.paho.org/hq/index.php?option=com_content&view=article&id=9657%3A2014-los-casos-dengue-americas-quintuplicaron-diez-anos-segun-nuevos-datos-opsoms&catid=740%3Anews-press-releases&Itemid=1926&lang=en

[2] Pan-American Health Organization/World Health Organization. Description of the current epidemiological trends of dengue in the Americas. [cited 2017 Jul 10]. http://www.paho.org/hq/index.php?option=com_content&view=article&id=4494&Itemid=2481&lang=en

[3] Boletín Epidemiología de la Ciudad. 2009 [cited 2016 Sep 19]. http://www.buenosaires.gob.ar/sites/gcaba/files/boletin-2009-en-linea.pdf

[4] Tittarelli E, Mistchenko AS, Barrero PR. Dengue virus 1 in Buenos Aires from 1999 to 2010: towards local spread. PLoS One. 2014;9:e111017. 10.1371/journal.pone.0111017 25343372 PMC4208802

[5] Virus Pathogen Database and Analysis Resource (ViPR). Flaviviridae—Metadata-driven Comparative Analysis Tool (meta-CATS) Report [cited 2016 Sep 9]. https://www.viprbrc.org/brc/mgc.spg?decorator=flavi&method=RetrieveResults&ticketNumber=MG_853990308837#

[6] Pinheiro FP. Dengue in the Americas. 1980-1987. Epidemiol Bull. 1989;10:1–8. 2484241

[7] Allicock OM, Lemey P, Tatem AJ, Pybus OG, Bennett SN, Mueller BA, et al. Phylogeography and population dynamics of dengue viruses in the Americas. Mol Biol Evol. 2012;29:1533–43. 10.1093/molbev/msr320 22319149 PMC3529620

[8] Villabona-Arenas CJ, Zanotto PMA. Worldwide spread of Dengue virus type 1. PLoS One. 2013;8:e62649. 10.1371/journal.pone.0062649 23675416 PMC3652851

[9] Tittarelli E, Barrero PR, Mistchenko AS, Valinotto LE. Secondary dengue virus infections during the 2009 outbreak in Buenos Aires. Trop Med Int Health. 2016;21:28–32. 10.1111/tmi.12619 26458156

[10] Chen R, Vasilakis N. Dengue—quo tu et quo vadis? Viruses. 2011;3:1562–608. 10.3390/v3091562 21994796 PMC3187692

[11] Drumond BP, Mondini A, Schmidt DJ, Bosch I, Nogueira ML. Population dynamics of DENV-1 genotype V in Brazil is characterized by co-circulation and strain/lineage replacement. Arch Virol. 2012;157:2061–73 https://www.ncbi.nlm.nih.gov/pubmed/22777179. 10.1007/s00705-012-1393-9 22777179

[12] Cunha MP, Guimarães VN, Souza M, de Paula Cardoso D, de Almeida TN, de Oliveira TS, et al. Phylodynamics of DENV-1 reveals the spatiotemporal co-circulation of two distinct lineages in 2013 and multiple introductions of dengue virus in Goiás, Brazil. Infect Genet Evol. 2016;43:130–4. 10.1016/j.meegid.2016.05.021 27223633

