# Supplementary material for: Dengue Virus 1 Outbreak in Buenos Aires, Argentina, 2016
Source: Emerg Infect Dis. 2017 Oct;23(10):1684–5. doi: 10.3201/eid2310.161718 (PMC5621538; doi:10.3201/eid2310.161718)
Supplement: Technical Appendix — Additional information about the sequencing and analysis of dengue virus genomes. [file 16-1718-Techapp-s1.pdf]

Article DOI: https://doi.org/10.3201/eid2310.161718

Dengue Virus 1 Outbreak in Buenos Aires, Argentina, 2016

Technical Appendix

Sequencing, analysis, and annotation

We extracted viral RNA from sera by standard EZ1 BioRobot protocol (EZ1 Virus Mini Kit v2.0; QIAGEN, Valencia, CA, USA) and stored it at –20°C. We amplified the coding sequence of the E protein using OneStep RT-PCR Kit (QIAGEN) according to the manufacturer's instructions. The primer pairs were 790: 5′-GGAGACTTGGGCTTTGCGACACCC-3′ / 1491: 5′-GCCCAGTTCTAGGTGAGCAG-3′; 1208: 5′-GTGGACAGAGGCTGGGGTAATGGC-3′ / 2257: 5′-GTCCAAGAAACACCGCTGAACA-3′ and 2125: 5′-AAGCAACCGCCCGAGGAG-3′ / 2904: 5′-GTAGGAGTCACGCAATTTCAACCA-3′, as previously described (*1*,*2*).

We analyzed sequences in an ABI3500 genetic analyzer and obtained consensus sequences by compiling overlapping reads with SeqScape Software v2.7 (Applied Biosystems, Foster City, CA, USA), reference sequence AF226687.2). We inferred amino acid sequences using the universal code by BioEdit software (*3*). The sequences were submitted to GenBank (KX768338-KX768419).

To determine how natural selection acted on the viruses analyzed in our laboratory, we measured the ratio of non-synonymous (dN) to synonymous (dS) substitutions per site (dN/dS) by Datamonkey and DNAsp v.5 (DNA Sequence Polymorphism) (*4*,*5*). The Datamonkey analysis was performed using the following codon-based maximum likelihood (ML) methods: Single Likelihood Ancestor Counting (SLAC), Fixed Effects Likelihood (FEL) and Random Effects Likelihood (REL) at the specified significance levels (p-value <0.1 and Bayes factor 50) (*6*). We used Meta-CATS statistical analysis tool available in NIAID Virus Pathogen Database and Analysis Resource (ViPR) online to identify amino acid positions that significantly differ between the sequences obtained in this study (*7*,*8*).

Genotyping

To genotype the sequences obtained from the 2016 outbreak, we included a total of 260 DENV sequences in the analysis. Of them, 82 sequences were obtained in this study, 27 DENV-1 sequences were previously reported by our laboratory and 134 sequences of different DENV-1 genotypes were retrieved from the NCBI Dengue Virus Resource (*9*). Sequences were aligned with MUSCLE (*10*).

We used jModelTest v.0.1.1 to determine that the GTR+G+I model was the appropriate nucleotide substitution model for the sequence alignment (*11*). We obtained phylogenetic inferences using Neighbor Joining (Mega software v.5.2.2 (*12*)), ML (PhyML v.20120412 software (*13*)) and Bayesian criteria (MrBayes software v.3.2.3 (*14*)). Branch support of the Neighbor Joining tree was evaluated by non-parametric bootstrapping with 1,000 pseudo-replica. We evaluated the convergence of Monte Carlo Markov Chains (MCMC) implemented in the Bayesian criteria with split frequencies ≤0.01 and in TRACER v.1.6 with an effective sample size (ESS) >200; the initial 10% of the run length was discarded as burn-in. We visualized consensus trees with FigTree v.1.4.3.

Phylodynamic and phylogeographic analyses

To evaluate the origin of the 2016 outbreak, we performed a discrete phylogeographic analysis on 198 E-protein sequences of American DENV-1 genotype V, including the 82 sequences obtained in this study. We associated each sequence to the country of probable infection and the year of collection. We used Bayesian coalescent-based methods implemented in the BEAST package v.1.8.2 (*15*). All BEAST run logs were analyzed with TRACER after evaluating the convergence of the MCMC as described in the previous section. The evolutionary model TIM3+I+G, the molecular clock Lognormal relaxed clock (uncorrelated), and the demographic model GMRF Bayesian Skyride used in BEAST package were selected by Bayes Factor. The maximum clade credibility tree was visualized by TreeAnnotator.

References

1. Barrero PR, Mistchenko AS. Complete genome sequencing of dengue virus type 1 isolated in Buenos Aires, Argentina. Virus Res. 2004;101:135–45. PubMed http://dx.doi.org/10.1016/j.virusres.2003.12.033

2. Zhang C, Mammen MPJr, Chinnawirotpisan P, Klungthong C, Rodpradit P, Monkongdee P, et al. Clade replacements in dengue virus serotypes 1 and 3 are associated with changing serotype prevalence. J Virol. 2005;79:15123–30. PubMed http://dx.doi.org/10.1128/JVI.79.24.15123-15130.2005

3. Hall TA. BioEdit: a user-friendly biological sequence alignment editor and analysis program for Windows 95/98/NT.Nucleic Acids Symp Ser. 1999;41:95–8.

4. Librado P, Rozas J. DnaSP v5: a software for comprehensive analysis of DNA polymorphism data. Bioinformatics. 2009;25:1451–2. PubMed http://dx.doi.org/10.1093/bioinformatics/btp187

5. Delport W, Poon AFY, Frost SDW, Kosakovsky Pond SL. Datamonkey 2010: a suite of phylogenetic analysis tools for evolutionary biology.Bioinformatics. 2010;26:2455–7. PubMed http://dx.doi.org/10.1093/bioinformatics/btq429

6. Kosakovsky Pond SL, Frost SDW. Not so different after all: a comparison of methods for detecting amino acid sites under selection. MolBiolEvol. 2005;22:1208–22. PubMed http://dx.doi.org/10.1093/molbev/msi105

7. Pickett BE, Sadat EL, Zhang Y, Noronha JM, Squires RB, Hunt V, et al. ViPR: an open bioinformatics database and analysis resource for virology research. Nucleic Acids Res. 2012;40(D1):D593–8. PubMed http://dx.doi.org/10.1093/nar/gkr859

8. Virus Pathogen Database and Analysis Resource (ViPR). Flaviviridae—Metadata-driven Comparative Analysis Tool (meta-CATS) Report [cited 2016 Sep 9]. https://www.viprbrc.org/brc/mgc.spg?decorator=flavi&method=RetrieveResults&ticketNumber=MG_853990308837#

9. National Center for Biotechnology Information. Database of single nucleotide polymorphisms (dbSNP) [cited 2013 Jan 7]. http://www.ncbi.nlm.nih.gov/snp

10. Edgar RC. MUSCLE: multiple sequence alignment with high accuracy and high throughput. Nucleic Acids Res. 2004;32:1792–7. PubMed http://dx.doi.org/10.1093/nar/gkh340

11. Darriba D, Taboada GL, Doallo R, Posada D. jModelTest 2: more models, new heuristics and parallel computing. Nat Methods. 2012;9:772. PubMed http://dx.doi.org/10.1038/nmeth.2109

12. Tamura K, Peterson D, Peterson N, Stecher G, Nei M, Kumar S. MEGA5: molecular evolutionary genetics analysis using maximum likelihood, evolutionary distance, and maximum parsimony methods. MolBiolEvol. 2011;28:2731–9. PubMed http://dx.doi.org/10.1093/molbev/msr121

13. Guindon S, Dufayard JF, Lefort V, Anisimova M, Hordijk W, Gascuel O. New algorithms and methods to estimate maximum-likelihood phylogenies: assessing the performance of PhyML 3.0. Syst Biol. 2010;59:307–21. PubMed http://dx.doi.org/10.1093/sysbio/syq010

14. Ronquist F, Huelsenbeck JP. MrBayes 3: Bayesian phylogenetic inference under mixed models. Bioinformatics. 2003;19:1572–4. PubMedhttp://dx.doi.org/10.1093/bioinformatics/btg180

15. Drummond AJ, Suchard MA, Xie D, Rambaut A. Bayesian phylogenetics with BEAUti and the BEAST 1.7. MolBiolEvol. 2012;29:1969–73. PubMed http://dx.doi.org/10.1093/molbev/mss075

Technical Appendix Table. Representative samples of DENV-1 collected from dengue patients during 2016 outbreak in Buenos Aires, Argentina

Sample	Collection date	Patient age, y/sex	Location of infection	Amino acid and position								
				180 A	222 S	235 D	325 K	338 S	361 K	394 R	428 V	436 V
HNRG102741	2016 Jan 19	36/M	Argentina, Misiones	–	–	–	–	–	–	–	–	–
HNRG106053	2016 Feb 29	65/F	Argentina, Buenos Aires	V	–	–	–	–	–	–	–	–
HNRG102917	2016 Jan21	7/F	Paraguay	V	–	–	–	–	–	–	–	–
HNRG106056	2016 Feb 29	10/M	Argentina, Buenos Aires	–	–	–	–	–	–	–	–	–
HNRG103674	2016 Feb 1	45/M	Argentina, Misiones	–	–	–	–	–	–	–	–	–
HNRG104323	2016 Feb 10	14/F	Argentina, Buenos Aires	–	–	–	–	–	–	–	–	–
HNRG104496	2016 Feb 11	63/F	Argentina, Buenos Aires	–	–	–	–	–	–	–	–	–
HNRG106613	2016 Mar 3	28/F	Brazil	–	–	–	–	–	–	–	–	–
HNRG106477	2016 Mar 2	39/M	Brazil	–	–	–	–	–	–	–	–	–
HNRG105181	2016 Feb 19	30/F	Argentina, Buenos Aires	–	–	–	–	–	–	–	–	–
HNRG104715	2016 Feb 15	31/F	Argentina, Buenos Aires	–	–	–	–	–	–	–	–	–
HNRG105546	2016 Feb 23	12/M	Argentina, Jujuy	–	–	–	–	–	–	–	–	–
HNRG105346	2016 Feb 22	18/F	Argentina, Buenos Aires	–	–	–	–	–	–	–	–	–
HNRG104228	2016 Feb 10	33/F	Argentina, Buenos Aires	–	–	–	–	–	–	–	–	–
HNRG105220	2016 Feb 19	31/F	Argentina, Buenos Aires	–	–	–	–	–	–	–	–	–
HNRG106428	2016 Mar 2	50/M	Argentina, Entre Ríos	–	–	–	–	–	–	–	–	–
HNRG104546	2016 Feb 12	28/F	Argentina, Buenos Aires	–	–	–	–	–	–	–	–	–
HNRG108046	2016 Mar 11	47/F	Argentina, San Luis	–	–	–	–	–	–	–	–	–
HNRG105079	2016 Feb 18	65/F	Argentina, Buenos Aires	–	–	–	–	–	–	–	–	–
HNRG105107	2016 Feb 18	41/M	Argentina, Buenos Aires	–	–	–	–	–	–	–	–	–
HNRG105184	2016 Feb 19	24/F	Argentina, Buenos Aires	–	–	–	–	–	–	–	–	–
HNRG104768	2016 Feb 15	45/F	Argentina, Buenos Aires	–	–	–	–	–	–	–	–	–
HNRG104789	2016 Feb 15	17/F	Argentina, Entre Ríos	–	–	–	–	–	–	–	–	–
HNRG107088	2016 Mar 7	32/F	Bolivia	–	–	–	–	–	–	–	–	–
HNRG107216	2016 Mar 7	30/M	Bolivia	–	–	–	–	–	–	–	–	–
HNRG105173	2016 Feb 19	43/F	Argentina, Buenos Aires	–	–	–	–	–	–	–	–	–
HNRG104763	2016 Feb 15	24/M	Argentina, Buenos Aires	–	–	–	–	–	–	–	–	–
HNRG104983	2016 Feb 17	18/F	Argentina, Buenos Aires	–	–	–	–	–	–	–	–	–
HNRG104743	2016 Feb 15	41/F	Argentina, Tucumán	–	–	–	–	–	–	–	–	–
HNRG105512	2016 Feb 23	33/F	Brazil	–	–	–	–	–	–	–	–	–
HNRG105620	2016 Feb 19	11/F	Brazil	–	–	–	–	–	–	–	–	–
HNRG105686	2016 Feb 24	35/F	Brazil	–	–	–	–	–	–	–	–	–
HNRG107304	2016 Mar 8	27/F	Argentina, Santiago del Estero	–	–	–	–	–	–	–	–	–
HNRG104502	2016 Feb 11	11/M	Argentina, Buenos Aires	–	–	–	–	–	–	–	–	–
HNRG102566	2016 Jan 14	1/M	Argentina, Misiones	–	–	–	–	–	–		–	–
HNRG106110	2016 Feb 29	25/F	Argentina, Buenos Aires	–	–	–	–	–	–	–	–	–
HNRG105949	2016 Feb 26	34/F	Brazil	–	T	–	–	–	–	–	–	–
HNRG105906	2016 Feb 26	43/F	Brazil	–	T	–	–	–	–	–	–	–

				Amino acid and position								
Sample	Collection date	Patient age, y/sex	Location of infection	180 A	222 S	235 D	325 K	338 S	361 K	394 R	428 V	436 V
HNRG105062	2016 Feb 18	40/F	Argentina, Buenos Aires	–	T	–	–	–	–	–	–	–
HNRG106045	2016 Feb 29	78/M	Argentina, Buenos Aires	–	T	–	–	–	–	–	–	–
HNRG104513	2016 Feb 11	52/M	Brazil	–	T	–	–	–	–	–	–	–
HNRG104075	2016 Feb 5	30/M	Brazil	–	T	–	–	–	–	–	–	–
HNRG104047	2016 Feb 4	35/M	Brazil	–	–	–	–	–	–	K	–	–
HNRG103430	2016 Jan 28	41/M	Argentina, Buenos Aires	–	–	E	R	L	R	K	L	I
HNRG103011	2016 Jan 22	29/M	Paraguay	–	–	E	R	L	R	K	L	I
HNRG104255	2016 Feb 10	30/M	Argentina, Buenos Aires	–	–	E	R	L	R	K	L	I
HNRG104580	2016 Feb 12	36/F	Argentina, Buenos Aires	–	–	E	R	L	R	K	L	I
HNRG103330	2016 Jan 27	51/F	Argentina, Misiones	–	–	E	R	L	R	K	L	I
HNRG103696	2016 Feb 1	26/F	Argentina, Buenos Aires	–	–	E	R	L	R	K	L	I
HNRG104100	2016 Feb 5	45/M	Argentina, Buenos Aires	–	–	E	R	L	R	K	L	I
HNRG108252	2016 Mar 11	42/M	Argentina, Misiones	–	–	E	R	L	R	K	L	I
HNRG105486	2016 Feb 23	39/F	Argentina, Buenos Aires	–	–	E	R	L	R	K	L	I
HNRG104247	2016 Feb 10	13/M	Argentina, Misiones	–	–	E	R	L	R	K	L	I
HNRG103745	2016 Feb 1	20/M	Brazil	–	–	–	–	L	–	K	L	I
HNRG107955	2016 Mar 10	13/F	Brazil	–	–	–	–	L	–	K	L	I
HNRG104048	2016 Feb 4	42/F	Argentina, Buenos Aires	–	–	–	–	L	–	K	L	I
HNRG108235	2016 Mar 11	52/M	Argentina, Formosa	–	–	–	–	L	–	K	L	I
HNRG104492	2016 Feb 11	29/F	Argentina, Buenos Aires	–	–	–	–	L	–	K	L	I
HNRG104842	2016 Feb 16	31/F	Argentina, Buenos Aires	–	–	–	–	L	–	K	L	I
HNRG104865	2016 Feb 16	43/M	Argentina, Buenos Aires	–	–	–	–	L	–	K	L	I
HNRG102652	2016 Jan 18	NA/M	Argentina, Formosa	–	–	–	–	L	–	K	L	I
HNRG106691	2016 Mar 3	52/M	Argentina, Córdoba	–	–	–	–	L	–	K	L	I
HNRG105776	2016 Feb 25	51/F	Argentina, Santiago del Estero	–	–	–	–	L	–	K	L	I
HNRG107038	2016 Mar 7	63/M	Brazil	–	–	–	–	L	–	K	L	I
HNRG106060	2016 Feb 29	23/F	Argentina, Buenos Aires	–	–	–	–	L	–	K	L	I
HNRG103677	2016 Feb 1	58/M	Argentina, Buenos Aires	–	–	–	–	L	–	K	L	I
HNRG105835	2016 Feb 25	19/F	Argentina, Santiago del Estero	–	–	–	–	L	–	K	L	I
HNRG105076	2016 Feb 18	13/M	Argentina, Buenos Aires	–	–	–	–	L	–	K	L	I
HNRG104922	2016 Feb 16	42/F	Argentina, Buenos Aires	–	–	–	–	L	–	K	L	I
HNRG104694	2016 Feb 15	12/M	Argentina, Buenos Aires	–	–	–	–	L	–	K	L	I
HNRG110386	2016 Apr 13	30/F	Argentina, Buenos Aires	–	–	–	–	L	–	K	L	I
HNRG106373	2016 Mar 1	49/F	Argentina, Chaco	–	–	–	–	L	–	K	L	I
HNRG105836	2016 Feb 25	39/M	Argentina, San Luis	–	–	–	–	L	–	K	L	I
HNRG105691	2016 Feb 24	28/M	Argentina, Entre Ríos	–	–	–	–	L	–	K	L	I
HNRG105481	2016 Feb 23	46/M	Argentina, Misiones	–	–	–	–	L	–	K	L	I
HNRG103646	2016 Feb 1	28/F	Argentina, Buenos Aires	–	–	–	–	L	–	K	L	I
HNRG102532	2016 Jan 14	16/M	Paraguay	–	–	–	–	L	–	K	L	I
HNRG103191	2016 Jan 25	43/F	Paraguay	–	–		–	L	–	K	L	I
HNRG105214	2016 Feb 19	63/M	Argentina, Buenos Aires	–	–	–	–	L	–	K	L	I
HNRG105212	2016 Feb 19	30/F	Argentina, Buenos Aires	–	–	–	–	L	–	K	L	I
HNRG107068	2016 Mar 7	42/F	Argentina, Córdoba	–	–	–	–	L	–	K	L	I
HNRG102886	2016 Jan 21	17/M	Argentina, Misiones	–	–	–	–	L	–	K	L	I

*Singleton variations are not shown. Letters indicate substitution, dashes no substitution. A, alanine; D, aspartic acid; E, glutamic acid; I, isoleucine; K, lysine; L, leucine; R, arginine; S, serine; T, threonine; V, valine; NA, not available.

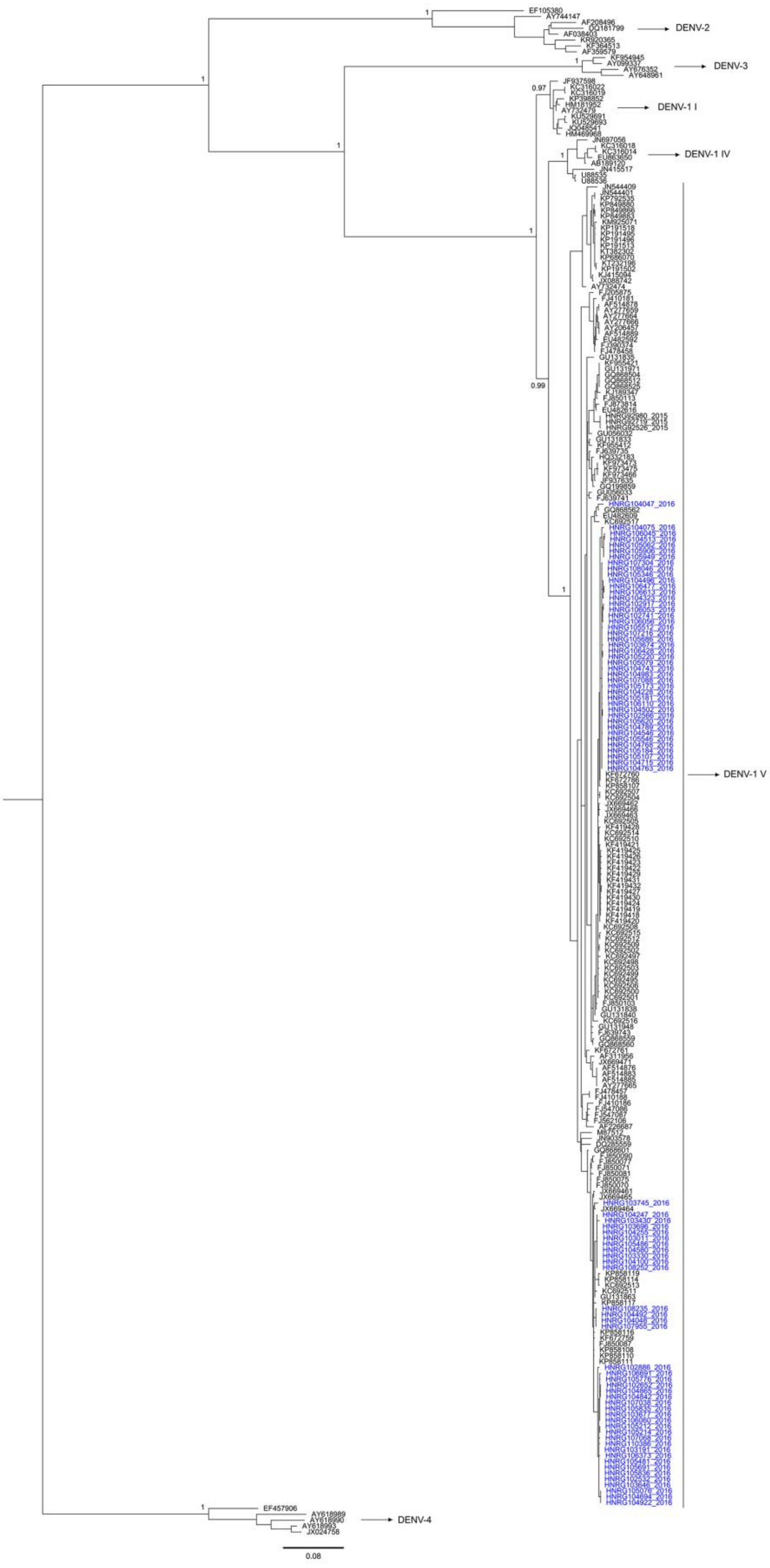
DENV-2
DENV-3
DENV-1 I
DENV-1 IV
DENV-1 V
DENV-4
0.08

Technical Appendix Figure 1. Phylogenetic Bayesian consensus tree. Posterior probabilities > 0.70 are shown on the nodes (4E+6 generations sampling every 4E+3 generations). Sequences included in the analysis are named with GenBank accession number. Sequences reported in this study are highlighted in blue.

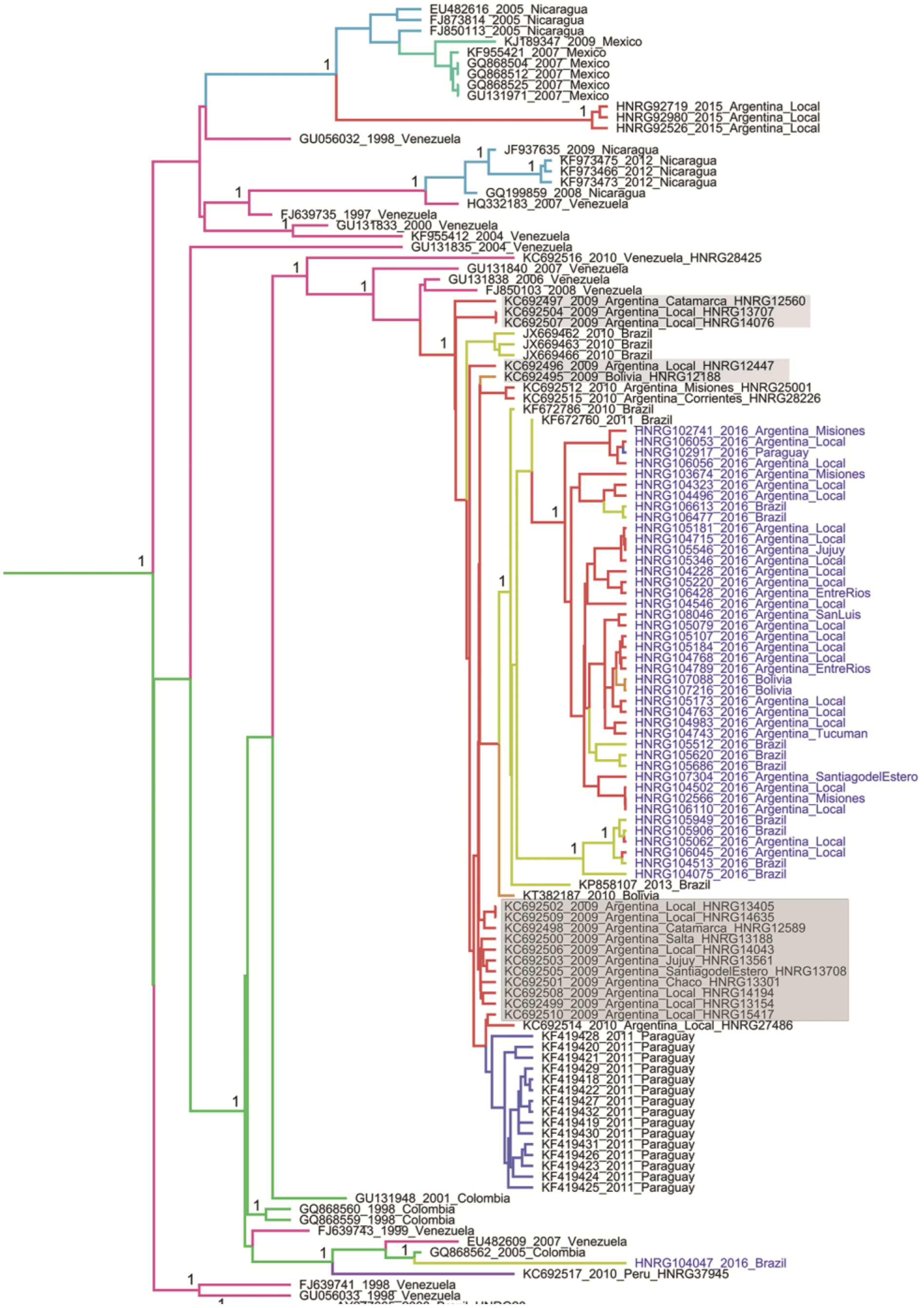

Technical Appendix Figure 2. Maximum clade credibility tree obtained by discrete phylogeographic analysis of the coding sequence from the envelope protein of dengue virus type 1 genotype V isolates

from the Americas. Posterior probabilities equal to 1 are shown on the nodes (2 x 108 generations sampling every 2 x 104 generations). Sequences downloaded from GenBank are named as accession number_year of collection_source country, and sequences obtained in the virology laboratory at Hospital de Niños R. Gutiérrez are named as HNRGnumber_year of collection_source country(_Argentinean province). Blue indicates sequences reported in this study; gray indicates sequences from 2009.
